# Effect of Dexmedetomidine on Posttraumatic Stress Disorder in Patients Undergoing Emergency Trauma Surgery: A Randomized Clinical Trial

**DOI:** 10.1001/jamanetworkopen.2023.18611

**Published:** 2023-06-16

**Authors:** Youjia Yu, Yan Li, Dan Han, Chuhao Gong, Liwei Wang, Beiping Li, Rui Yao, Yangzi Zhu

**Affiliations:** 1 Department of Anesthesiology, Suzhou Xiangcheng People’s Hospital, Suzhou, China; 2 Department of Anesthesiology, Xuzhou Renci Hospital, Xuzhou, China; 3 Department of Anesthesiology, Xuzhou Central Hospital, Xuzhou, China; 4 Department of Anesthesiology, Xuzhou First People’s Hospital, Xuzhou, China; 5 Jiangsu Province Key Laboratory of Anesthesiology, Xuzhou Medical University, Xuzhou, China

## Abstract

**Importance:**

Posttraumatic stress disorder (PTSD) is common in people who have experienced trauma, especially those hospitalized for surgery. Dexmedetomidine may reduce or reverse the early consolidation and formation of conditioned fear memory and prevent the occurrence of postoperative PTSD.

**Objective:**

To evaluate the effects of intraoperative and postoperative low-dose intravenous pumping dexmedetomidine on PTSD among patients with trauma undergoing emergency surgery.

**Design, Setting, and Participants:**

This double-blind, randomized clinical trial was conducted from January 22 to October 20, 2022, with follow-up 1 month postoperatively, in patients with trauma undergoing emergency surgery at 4 hospital centers in Jiangsu Province, China. A total of 477 participants were screened. The observers were blinded to patient groupings, particularly for subjective measurements.

**Interventions:**

Dexmedetomidine or placebo (normal saline) was administered at a maintenance dose of 0.1 μg/kg hourly from the start of anesthesia until the end of surgery and at the same rate after surgery from 9 pm to 7 am on days 1 to 3.

**Main Outcomes and Measures:**

The primary outcome was the difference in the incidence of PTSD 1 month after surgery in the 2 groups. This outcome was assessed with the Clinician-Administered PTSD Scale for *Diagnostic and Statistical Manual of Mental Disorders* (Fifth Edition) (CAPS-5). The secondary outcomes were the pain score within 48 hours and 1 month postoperatively; incidence of postoperative delirium, nausea, and pruritus; subjective sleep quality; anxiety; and occurrence of adverse events.

**Results:**

A total of 310 patients (154 in the normal saline group and 156 in the dexmedetomidine group) were included in the modified intention-to-treat analysis (mean [SD] age, 40.2 [10.3] years; 179 men [57.7%]). The incidence of PTSD was significantly lower in the dexmedetomidine group than in the control group 1 month postoperatively (14.1% vs 24.0%; *P* = .03). The participants in the dexmedetomidine group had a significantly lower CAPS-5 score than those in the control group (17.3 [5.3] vs 18.9 [6.6]; mean difference, 1.65; 95% CI, 0.31-2.99; *P* = .02). After adjusting for potential confounders, the patients in the dexmedetomidine group were less likely to develop PTSD than those in the control group 1 month postoperatively (adjusted odds ratio, 0.51; 95% CI, 0.27-0.94; *P* = .03).

**Conclusions and Relevance:**

In this randomized clinical trial, the administration of intraoperative and postoperative dexmedetomidine reduced the incidence of PTSD among patients with trauma. The findings of this trial support the use of dexmedetomidine in emergency trauma surgery.

**Trial Registration:**

Chinese Clinical Trial Register Identifier: ChiCTR2200056162

## Key Points

**Question:**

Does perioperative administration of low-dose dexmedetomidine have a protective effect on posttraumatic stress disorder in patients with trauma undergoing emergency surgery?

**Finding:**

In this randomized clinical trial of 310 patients, posttraumatic stress disorder occurred in 14.1% of patients in the dexmedetomidine group and 24.0% of patients in the control group 1 month postoperatively.

**Meaning:**

The findings of this study support the perioperative use of dexmedetomidine for reducing the incidence of posttraumatic stress disorder in patients with trauma undergoing emergency surgery.

## Teaser

This randomized clinical trial investigates the effects of dexmedetomidine on the incidence of postoperative posttraumatic stress in patients with trauma undergoing emergency surgery.

## Introduction

Posttraumatic stress disorder (PTSD) is a psychiatric disorder that develops after experiencing major trauma^1^ and involves a combination of recurring and distressing reexperiencing (eg, flashbacks and intrusive thoughts), avoidance, negative alterations in mood and cognition, and hyperarousal. Similar or related stimuli may persist for years or decades, with recurrent episodes of traumatic experiences and sustained increases in vigilance and avoidance.^2,3,4^ This mental state is highly debilitating and severely interferes with daily life and social activities.^5,6^ In recent years, the global incidence of PTSD has reached rates up to 10% to 22%, revealing a significant upward trend because of the frequent occurrence of traffic accidents, natural disasters, wars, and terrorist violence.^7,8,9^ Studies have shown that the prevalence of PTSD in the general US population is 6% to 8%, and it can be as high as 13% to 30% in the military.^10,11^ The incidence of PTSD after trauma hospitalization can be as high as 23%.^12^ Once PTSD is formed, it can be difficult to manage and is linked to increased risk of suicide, posing a serious burden to families and society.^7,13,14^ Therefore, early and timely intervention for patients with trauma is particularly critical to prevent PTSD.

The pathogenesis of PTSD is complex, and its exact neurobiological mechanism is still unclear.^15^ Studies have shown that recurrent traumatic experience is a core symptom of PTSD, which is closely related to abnormally strengthened fear memory.^16,17^ Because of the pavlovian conditioning principle, environmental information at the time of trauma (eg, loud sounds or objects) is associated with the aversive experience (eg, accident or fall injury). The reexposure of wounded persons to a similar environment may bring back fear memory and lead to physiologic and behavioral reactions, which is called fear conditioning.^18^ Fear conditioning is an outstanding memory feature of PTSD that can explain reexperiencing and, in part, avoidance symptoms.^19^ Therefore, intervening in the consolidation and formation of conditioned fear memory is particularly critical to prevent PTSD in patients with trauma in the emergency department, who are in the early stage of fear memory formation and are not yet firmly consolidated.

Dexmedetomidine is widely used in clinical anesthesia to optimize anesthesia and analgesia effects and reduce intraoperative adverse reactions.^20^ A preclinical study shows that dexmedetomidine could alleviate anxiety-like behavior and cognitive impairment in PTSD model rats.^21^ In clinical studies, the perioperative administration of dexmedetomidine had an anxiolytic effect.^22,23^ In another study of conditioned fear memory, dexmedetomidine reduced the strength of the fear memory formed.^24^ Therefore, we speculate that dexmedetomidine might attenuate the formation and consolidation of conditioned fear memories early in trauma, thereby preventing the development of PTSD. However, whether dexmedetomidine can reduce the incidence of postoperative PTSD in patients with trauma in the emergency department is still unclear. This study aimed to investigate the effects of dexmedetomidine on the incidence of postoperative PTSD in patients with trauma in the emergency department to lay a theoretical foundation for finding a more effective preventive measure for such patients.

## Methods

### Study Design and Population

This multicenter, double-blind, randomized clinical trial was conducted at Suzhou Xiangcheng People’s Hospital and 3 other tertiary hospitals in Jiangsu Province, China, from January 22 to October 20, 2022. The study protocol was approved by the ethics committees of all participating hospitals and registered in the Chinese Clinical Trial Registratiy Center on January 21, 2022. Written informed consent was obtained from all patients or their families. This report follows the Consolidated Standards of Reporting Trials (CONSORT) reporting guideline for randomized studies. The full trial protocol is available in Supplement 1.

### Study Procedures and Data Collection

Our research team identified potential participants in the emergency department via the emergency clinical information system (Maidi Technology Co., Ltd). The system contained the patient’s medical record information and the time to call the emergency number. The time from calling the emergency number to anesthesia induction was used as the trauma time in this study. Acute Physiology and Chronic Health Evaluation II (APACHE II)^25^ and Injury Severity Score (ISS)^26^ were used to assess the severity of trauma. Baseline data were collected, the emergency channel was started, and the emergency operating room was informed in advance. Eligible participants were randomized to the dexmedetomidine or normal saline placebo group. The study medication (dexmedetomidine hydrochloride, 200 μg/2 mL, and normal saline, 2 mL) was provided and assigned on the basis of randomization by an anesthetic nurse who was not involved in the rest of the study. The drug was diluted to 50 mL with normal saline before administration (ie, dexmedetomidine hydrochloride at a final concentration of 4 μg/mL). Dexmedetomidine or placebo (normal saline) was administered at a maintenance dose of 0.1 μg/kg hourly from the start of anesthesia until the end of surgery and at the same rate after surgery from 9 pm to 7 am on days 1 to 3. The infusion of dexmedetomidine or placebo was suspended or permanently stopped during or after the operation according to the patient’s own condition or other objective factors. The dose of infusion was recorded. Blood pressure, heart rate, and pulse oxygen saturation were measured 3 days after the operation. The perioperative clinical data, intensive care unit (ICU) admission, and perioperative adverse events were recorded.

### Participants

The CONSORT flowchart is shown in the Figure. Patients with trauma (eg, car crash, falling, engineering incident, and so on) undergoing emergency surgery, aged 18 to 60 years, and with American Society of Anesthesiologists (ASA) physical status categories I (physical health, good development and nutrition, normal organ function), II (besides the surgical disease, there were mild coexisting conditions and sound functional compensation), or III (severe comorbidity, limited physical activity, but able to cope with daily activities) were eligible for inclusion. A total of 477 participants were screened. The exclusion criteria were as follows: (1) craniocerebral or spinal cord injury and hemorrhagic shock decompensation; (2) liver or kidney dysfunction; (3) history of alcohol abuse or drug dependence or history of neurologic or psychiatric diseases; (4) severe visual, hearing, or language impairment; (5) history of major physical or mental trauma; and (6) second-degree or third-degree heart blockage or bradyarrhythmia with a baseline heart rate lower than 50 beats/min.

**Figure.**
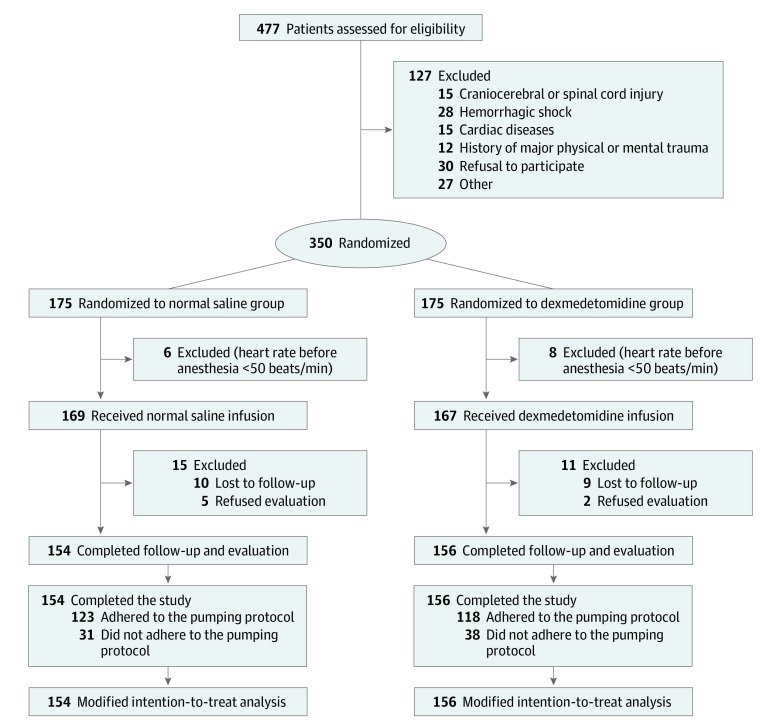
CONSORT Flow Diagram Dexmedetomidine was used in the dexmedetomidine group. The maintenance dose was 0.1 μg/kg per hour from the beginning of anesthesia until the end of surgery administered between 9 pm and 7 am on postoperative days 1 to 3. Patients in the control group received equivalent volumes of normal saline following the same protocol.

### Randomization and Blinding

Eligible participants were randomly assigned to dexmedetomidine or normal saline placebo (control group) using a 1:1 ratio by an online central randomization system. The randomization sequence was based on computer-generated random numbers. Patient clinical management and data collection were sequentially numbered and disclosed by health care practitioners who were not directly involved in this study. Each code was assigned by a random number to 1 of the 2 groups: the placebo group or the dexmedetomidine group. An anesthetic nurse who was not otherwise involved in the study prepared dexmedetomidine and normal saline in advance, which were each kept in syringes labeled only with the patient number. The surgeons, study statistician personnel, research staff who assessed the outcomes, and patients themselves were blinded to the treatment group.

### Anesthesia

The intraoperative monitoring included noninvasive blood pressure, electrocardiography, pulse oxygen saturation, radial artery blood pressure, and nasopharyngeal temperature. The intravenous anesthesia induction was performed using midazolam, sufentanil, etomidate, and rocuronium. After successful tracheal intubation, the patient received mechanical ventilation and end-tidal carbon dioxide was maintained between 35 and 45 mm Hg. Anesthesia was maintained using propofol, remifentanil, and cisatracurium to maintain a bispectral index value of 40 to 60 (index ranges from 1 to 100; higher values indicate shallower depths of sedation). Hypotension (mean arterial pressure <65 mm Hg or a decrease of 20% from baseline) and bradycardia (heart rate <50 beats/min) were treated. A thermal blanket was used during the operation to maintain a nasopharyngeal temperature above 36 °C. Patient-controlled intravenous analgesia was used in both groups. Postoperative analgesia was achieved with 3 μg/kg of sufentanil and 20 mg of azasetron in 100 mL of normal saline. The background infusion rate was 2 mL/h. After returning to the ward, if the visual analog scale (VAS) pain score was greater than 4 (range, 0-10; higher scores indicating more severe pain), 100 mg of intravenous flurbiprofen axetil was administered.

### Outcome Measure

The primary outcome was the occurrence of PTSD, assessed with the Clinician-Administered PTSD Scale for *Diagnostic and Statistical Manual of Mental Disorders* (Fifth Edition) (CAPS-5) 1 month after surgery. The CAPS-5 score (derived from the CAPS-5 scale) was used to evaluate PTSD severity. Professionally trained physicians, who were blinded to treatment group assignments, performed the diagnostic assessments at both times in tranquil surroundings. The CAPS-5 is a structured diagnostic interview and is considered the criterion standard in PTSD evaluation. The CAPS-5 provides a continuous measure of the severity of overall PTSD and of the 4 symptom clusters (intrusions, avoidance, negative alterations in cognition or mood, and arousal and reactivity) and the presence or absence of PTSD diagnosis, which can be administered by appropriately trained paraprofessionals.^27^ The diagnosis requirement can be summarized as an exposure to a stressor that is accompanied by at least 1 intrusion symptom, 1 avoidance symptom, 2 negative alterations in cognitions and mood symptoms, and 2 arousal and reactivity turbulence symptoms, persisting for at least 1 month, with functional impairment.^28^ The secondary outcomes included postoperative (24 hours, 48 hours, and 1 month) pain using the VAS; postoperative delirium using the confusion assessment method criteria^29^ (measured twice daily for 3 days); nausea, pruritus, or subjective sleep quality (measured for 3 days) using the numerical rating scale (scores range from 0 to 10, with 0 indicating the best and 10 the worst sleep quality); anxiety (measured for 3 days) using the Beck Anxiety Inventory (scores range from 0 to 84, with higher scores indicating increased anxiety); and occurrence of adverse events (including hypertension, hypotension, bradycardia, tachycardia, hypoxemia, and other complications, such as cerebrovascular events, myocardial infarction, heart failure, and acute kidney injury).

### Statistical Analysis

According to the literature, the incidence of PTSD among patients with trauma 1 month postoperatively was 23.2%.^30^ Our pilot study showed that 11.1% of patients with trauma in the emergency department who received dexmedetomidine during surgery developed PTSD 1 month postoperatively. Hence, this trial required 152 patients in each group with a power of 80% at a significance of α = .05. We decided to recruit 350 patients (with 175 in each group), considering a possible dropout rate of 15%.

The outcome analyses were performed in the modified intention-to-treat population. The data were analyzed using SPSS statistical software, version 23.0 (IBM Inc). The Kolmogorov-Smirnov test was used to determine whether the continuous data conformed to the normal distribution. Continuous variables were presented as the means (SDs) or medians (IQRs). The continuous data with normal distribution were compared with the independent-sample, 2-tailed, unpaired *t* test. The continuous data with nonnormal distribution were analyzed using the Kruskal-Wallis rank-sum test. Associations between categorical variables were assessed using the χ^2^ test or Fisher exact test. The odds ratios (ORs) and 95% CIs were calculated to analyze the effect of dexmedetomidine on the prevention of PTSD in the primary outcome. The association between the primary outcome and intervention was adjusted for some potential confounders using binary logistic regression, including age, sex, smoking, trauma time, ISS, APACHE II, ICU admission, type of surgery, study sites, and duration of surgery. The ORs and 95% CIs were calculated to analyze the association between dexmedetomidine dose and occurrence of PTSD in the post hoc analyses. The association between dexmedetomidine dose and occurrence of PTSD was adjusted for some potential confounders using binary logistic regression, including age, sex, smoking, trauma time, ISS, APACHE II, ICU admission, type of surgery, study sites, and duration of surgery. Repeated measures of continuous variables at different times in secondary outcomes were analyzed using repeated-measures analysis of variance. The Spearman correlation test was used to analyze the correlation between dexmedetomidine dose and CAPS-5 score in the post hoc analysis. A 2-sided *P* < .05 indicated a statistically significant association.

## Results

### Baseline Characteristics and Perioperative Data

Of 477 participants screened, 350 eligible patients were enrolled. The participant demographic characteristics and general clinical history in the 2 groups are given in eTable 1 in Supplement 2. Of these patients, 336 received the allocated intervention, and 310 patients completed the follow-up and evaluation (Figure). The remaining 310 patients (154 in the normal saline group and 156 in the dexmedetomidine group) were included in the modified intention-to-treat analysis, including 179 men (57.7%) and 131 women (42.3%), with a mean (SD) age of 40.2 (10.3) years. The participant demographic characteristics and general clinical history in the 2 groups are given in Table 1.

**Table 1.** Patient Characteristics and Perioperative Data^a^

Characteristic	Normal saline (n = 154)	Dexmedetomidine (n = 156)	*P* value
Sex			
Female	62 (40.3)	69 (44.2)	.48
Male	92 (59.7)	87 (55.8)	
Age, mean (SD), y	39.2 (10.4)	41.2 (10.0)	.09
BMI, mean (SD)	25.0 (3.6)	25.9 (4.9)	.09
American Society of Anesthesiologists physical status category			
I	108 (70.1)	98 (62.8)	.27
II	27 (17.5)	39 (25)	
III	19 (12.3)	19 (12.2)	
Hypertension	28 (18.2)	19 (12.2)	.14
Diabetes	18 (11.7)	23 (14.7)	.43
Smoking	32 (20.8)	28 (17.9)	.53
APACHE II score, median (IQR)	7.3 (5.7-9.4)	7.0 (5.0-9.0)	.42
ISS, median (IQR)	14.2 (6.8-19.9)	13.5 (8.7-19.7)	.76
Anatomical site of surgery			
Intestines	40 (26.0)	29 (18.6)	.18
Liver or spleen	17 (11.0)	29 (18.6)	
Limb	75 (48.7)	75 (48.1)	
Lungs, ribs, or diaphragm	22 (14.3)	23 (14.7)	
Trauma time, median (IQR), min^b^	100 (85-120)	95 (90-120)	.63
Surgery time, median (IQR), min	90 (80-120)	95 (71-120)	.83
Delayed emergence from anesthesia	7 (4.5)	12 (7.7)	.25
Intraoperative awareness			
Blood loss, median (IQR), mL	525 (275-700)	500 (300-600)	.73
Blood transfusion	29 (18.8)	26 (16.7)	.62
ICU admission	15 (9.7)	14 (9.0)	.82

Abbreviations: APACHE II, Acute Physiology and Chronic Health Evaluation II; BMI, body mass index (calculated as weight in kilograms divided by height in meters squared); ICU, intensive care unit; ISS, Injury Severity Score.

^a^ Data are presented as number (percentage) of patients unless otherwise indicated.

^b^ Trauma time was the time from calling the emergency to anesthesia induction.

### Primary Outcome

The dexmedetomidine group had a significantly lower PTSD incidence compared with the normal saline group (22 of 156 patients [14.1%] vs 37 of 154 patients [24.0%]; adjusted OR, 0.51; 95% CI, 0.27-0.94; *P* = .03). The dexmedetomidine group had a significantly lower CAPS-5 score compared with the normal saline group (17.3 [5.3] vs 18.9 [6.6]; mean difference, 1.65; 95% CI, 0.31-2.99; *P* = .02). Those in the dexmedetomidine group were less likely to screen for PTSD compared with those in the saline group (unadjusted OR, 0.52; 95% CI, 0.29-0.93; *P* = .03) even after adjusting for age, sex, smoking, trauma time, ISS, APACHE II, ICU admission, type of surgery, study sites, and duration of surgery.

### Secondary Outcomes

Table 2 gives the clinical postoperative outcomes. No significant differences were observed in VAS for pain 24 hours, 48 hours, and 1 month postoperatively and the occurrence of delirium, nausea, and pruritus within 3 days postoperatively in the 2 groups. The numerical rating scale for subjective sleep quality scores on the first, second, and third mornings postoperatively were all lower in the dexmedetomidine group than in the normal saline group. The Beck Anxiety Inventory for anxiety score on the first, second, and third mornings postoperatively were all lower in the dexmedetomidine group than in the normal saline group.

**Table 2.** Clinical Postoperative Outcomes Among the 2 Study Groups

Outcome	Normal saline (n = 154)	Dexmedetomidine (n = 156)	Difference or OR (95% CI)	*P* value
VAS for postoperative pain score, mean (SD)				
25 h^a^	3.2 (1.3)	2.9 (1.4)	0.23 (−0.07-0.54)^b^	.13
48 h^a^	1.9 (0.8)	1.7 (0.9)	0.16 (−0.03-0.35)^b^	.10
1 mo^a^	0 (0-2)	0 (0-2)	0.00 (0.00-0.00)^c^	.86
Adverse effects, No. (%) of patients				
Delirium	23 (14.9)	20 (12.8)	1.17 (0.67-2.03)^d^	.59
Nausea	23 (14.9)	27 (17.3)	0.86 (0.52-1.44)^d^	.57
Pruritus	16 (10.4)	11 (7.1)	1.47 (0.71-3.07)^d^	.30
NRS score for postoperative subjective sleep quality, median (IQR)				
First morning^e^^,^^f^	3 (2-5)	2 (1.25-3)	1.21 (0.84-1.58)^b^	<.001
Second morning^e^^,^^f^	2 (2-4)	2 (1-3)	1.04 (0.70-1.39)^b^	<.001
Third morning^e^^,^^f^	2 (2-4)	2 (1-2)	0.88 (0.57-1.19)^b^	<.001
BAI postoperative scores, median (IQR)				
First morning^f^^,^^g^	14 (10-18)	12 (8-17.75)	1.69 (0.26-3.13)^b^	.02
Second morning^f^^,^^g^	12 (10-18)	11 (8-15)	1.76 (0.50-3.03)^b^	.01
Third morning^f^^,^^g^	12 (8-6)	10 (8-15)	1.76 (0.64-2.89)	.002

Abbreviations: BAI, Beck Anxiety Inventory; NRS, numerical rating scale; OR, odds ratio; VAS, visual analog scale.

^a^ The VAS pain score ranged from 0 to 10, with 0 indicating no pain and 10 indicating most severe pain.

^b^ Difference in means.

^c^ Difference in medians.

^d^ OR.

^e^ The NRS for subjective sleep quality score ranged from 0 to 10, with 0 indicating the best and 10 the worst sleep quality.

^f^ Repeated test analysis of variance was used.

^g^ The BAI score ranged from 0 to 84, with higher scores indicating increased anxiety.

### Perioperative Adverse Events

The incidence of hypertension, tachycardia, hypotension, bradycardia, and hypoxemia during and after the procedure was not significantly different between the dexmedetomidine and normal saline group (eTable 2 in Supplement 2). None of the patients with trauma developed postoperative stroke, myocardial infarction, acute kidney injury, or heart failure.

### Post Hoc Analyses

Because of a high nonadherence rate, we performed a post hoc analysis of participants who complied with the pumping protocol to compare the incidence of PTSD between the 2 groups (as per-protocol analysis). The results were similar to those of the initial analysis (eFigure 1 in Supplement 2). We also performed post hoc analyses of the association between dexmedetomidine dose and occurrence of PTSD and CAPS-5 score in the intervention group. After adjusting for age, sex, smoking, trauma time, ISS, APACHE II, ICU admission, type of surgery, study sites, and duration of surgery, the dexmedetomidine dose was found to be an influencing factor for PTSD (adjusted OR, 0.90; 95% CI, 0.82-0.99; *P* = .02) (eTable 3 in Supplement 2). Spearman analysis was performed on the correlation between dexmedetomidine dose and CAPS-5 score in the dexmedetomidine group. The results showed no significant correlation between dexmedetomidine dose and the CAPS-5 score in the dexmedetomidine group (*r* = –0.07, *P* = .36) (eFigure 2 in Supplement 2).

## Discussion

Posttraumatic stress disorder is arguably the most common psychiatric disorder to arise after exposure to a traumatic event. Although trauma-focused cognitive behavior therapy is the best-validated treatment for PTSD, it has stagnated during recent decades, and only two-thirds of patients with PTSD respond adequately to this intervention. Moreover, most people with PTSD do not access evidence-based treatment.^31^ Therefore, preventing the occurrence of PTSD for high-risk populations in certain situations, such as emergency surgery for patients with trauma, is particularly urgent. This trial was the first, to our knowledge, to show the effect of perioperative dexmedetomidine administration on the prevention of PTSD.

In this randomized clinical trial, the patients with trauma who were sedated with dexmedetomidine were less likely to develop PTSD compared with those infused with normal saline. The trial found that the occurrence of PTSD in patients with trauma in the emergency department could be prevented by early anesthetic management. Our results showed that the CAPS-5 scores and the incidence of PTSD were significantly lower in the dexmedetomidine group compared with the control group 1 month after surgery, indicating that dexmedetomidine could reduce the severity and occurrence of PTSD in patients with trauma in the emergency department. However, the occurrence of PTSD is affected by a variety of factors.^30,32,33,34,35^ After adjusting for some potential confounders, perioperative pumping dexmedetomidine was a protective factor for PTSD. These results provided further evidence that dexmedetomidine could prevent the development of postoperative PTSD in patients with trauma in the emergency department.

We performed a post hoc subanalysis of patients with the intervention because of a high nonadherence rate in the intervention group. In our study, dexmedetomidine dose was negatively correlated with the occurrence of PTSD in the dexmedetomidine group. Dose and time window effects of dexmedetomidine in preventing the occurrence of PTSD might exist in patients undergoing emergency trauma surgery. The study found no significant correlation between dexmedetomidine dose and the CAPS-5 scores in the dexmedetomidine group. No relationship was found between PTSD severity and dexmedetomidine dose. A ceiling effect of the total dose of dexmedetomidine might exist, or the sample size of this subgroup analysis was not enough to detect the difference. Further studies are needed to confirm the exact relationship between dexmedetomidine dose and PTSD.

Hemodynamic changes are of concern in patients with trauma sedated with dexmedetomidine. Dexmedetomidine inhibits central sympathetic excitation because of its sedative effect, thereby reducing the patient’s heart rate and mean arterial blood pressure. However, dexmedetomidine can also activate peripheral α_2_-adrenergic receptors, leading to elevated mean arterial pressure.^36,37^ Our study showed that the extent of hypotension or hypertension, bradycardia, and tachycardia was similar to that in the control group. The results suggested that low-dose dexmedetomidine pumped during and after emergency trauma surgery did not cause circulatory instability. This finding might be attributable to the relatively conservative dosing of dexmedetomidine pumped in this study and the relatively strict exclusion criteria that excluded patients with circulatory instability, such as patients with decompensated hemorrhagic shock and elderly patients. Although patients with trauma who experienced decompensated shock were excluded from this study, still some critically ill patients with trauma were in the compensatory period. Our results indicate that the low-dose dexmedetomidine chosen in this study based on a previous study^38^ was safe.

In addition to dexmedetomidine, other anesthetics used during the surgery also had potential effects on PTSD. Previous studies found that propofol had an unusual reinforcing effect on conditioned fear memory and increased the risk of postoperative PTSD.^24,30,39^ In our study, the confounding factors were well controlled, and intravenous general anesthesia was used uniformly. Propofol is currently the most commonly used intravenous anesthetic drug in clinical anesthesia because of its rapid onset, convenient use, short half-life, and rapid recovery.^40^ Patients with physical trauma from traumatic events, such as car crashes, earthquakes, and falls, often require early emergency surgical treatment or ICU sedation. Sometimes they inevitably need to be sedated with propofol. Therefore, propofol intravenous general anesthesia was used in our study. Our results suggest that dexmedetomidine administration might be a beneficial choice when using propofol in patients with trauma undergoing emergency surgery.

The pathogenesis of PTSD is complex, and several potential mechanisms may underlie the preventive effects of dexmedetomidine on PTSD. The release of norepinephrine during or shortly after stressful and traumatic events can increase the formation of event-related fear memory, thereby inducing PTSD.^41^ Dexmedetomidine is a potent and highly selective α_2_-adrenergic receptor agonist, which mainly produces action in the locus coeruleus.^42^ Dexmedetomidine can reduce the consolidation, reinforcement, and formation of conditioned fear memory in the early stage of trauma by reducing the important source of norepinephrine in the central nervous system, so as to prevent the development of PTSD.

Notably, dexmedetomidine had no effect on the incidence of postoperative delirium after 3 days and pain scores after 2 days post surgery. This finding might be closely related to the relatively young patients with trauma in this study, time and dose of dexmedetomidine used, sample capacity, and relatively well-developed postoperative analgesia. Sleep quality is closely related to PTSD.^43^ A previous study showed that improving sleep quality could moderate PTSD symptoms.^44^ In the current study, low-dose dexmedetomidine significantly improved the sleep quality of patients with trauma in the emergency department for several days after the surgery, which may be one of the important reasons why dexmedetomidine prevented PTSD. Anxiety disorder shares neurobiological features with PTSD and is highly comorbid with PTSD.^45^ The current study found that dexmedetomidine significantly reduced postoperative anxiety scores in patients with trauma, which might be another important reason why dexmedetomidine prevented PTSD.

### Limitations

This study has several limitations that deserve mention. First, the 8.3% of patients unavailable during the 1-month follow-up might have resulted in statistical limitations of the PTSD incidence. Second, the population in this study was relatively narrow because of the use of strict inclusion and exclusion criteria. However, the strict inclusion and exclusion criteria in this study were based on a combination of safety and interference factors. Third, a previous study suggested that PTSD was associated with chronic pain.^46^ Although we assessed pain scores within 2 days and 1 month postoperatively, the lack of data on chronic postsurgical pain was still a limitation in this study. Fourth, this study investigated the effects of dexmedetomidine on postoperative PTSD only in patients with trauma under intravenous general anesthesia in the emergency department. Hence, the effects under other anesthesia modalities need to be further studied. Fifth, a few people were excluded from the study for various reasons. Nevertheless, their mental health remains noteworthy after surgery. We will attempt to obtain the neuropsychological data from these excluded populations in the long-term follow-up. Sixth, this study evaluated patients with trauma in the emergency department after only 1 month, and a systematic review of traumatic brain injury showed changes in the prevalence of PTSD after 3, 6, 12, and 24 months.^47^ Our follow-up studies will extend the duration of follow-up.

## Conclusions

In this randomized clinical trial, the administration of intraoperative and postoperative dexmedetomidine reduced the incidence of PTSD among patients with trauma. The findings of this trial support the use of dexmedetomidine in emergency trauma surgery. Therefore, we recommend low-dose dexmedetomidine sedation during and after surgery for patients with trauma under suitable conditions.

## Notes

Supplement 1.
Trial Protocol

Supplement 2. **eTable 1.** Patient Characteristics and Perioperative Data **eTable 2.** Safety Outcomes Among the Two Groups **eTable 3.** Association Between Dexmedetomidine Dose and Occurrence of PTSD in the Dexmedetomidine Group **eFigure 1.** Post Hoc Analysis of Participants Who Were Compliant With Pumping Protocol to Compare the Incidence of PTSD Among 2 Groups **eFigure 2.** Relationship Between Dexmedetomidine Dose and CAPS-5 Score

Supplement 3.
Data Sharing Statement

## References

[1] Hori H, Kim Y. Inflammation and post-traumatic stress disorder. Psychiatry Clin Neurosci. 2019;73(4):143-153. doi:10.1111/pcn.12820 30653780

[2] Koirala R, Søegaard EGI, Thapa SB. Updates on pharmacological treatment of post-traumatic stress disorder. JNMA J Nepal Med Assoc. 2017;56(206):274-280. doi:10.31729/jnma.3108 28746330

[3] He M, Wei JX, Mao M, . Synaptic plasticity in PTSD and associated comorbidities: the function and mechanism for diagnostics and therapy. Curr Pharm Des. 2018;24(34):4051-4059. doi:10.2174/1381612824666181120094749 30457048

[4] Qi W, Gevonden M, Shalev A. Prevention of post-traumatic stress disorder after trauma: current evidence and future directions. Curr Psychiatry Rep. 2016;18(2):20. doi:10.1007/s11920-015-0655-0 26800995 PMC4723637

[5] McCleery JM, Harvey AG. Integration of psychological and biological approaches to trauma memory: implications for pharmacological prevention of PTSD. J Trauma Stress. 2004;17(6):485-496. doi:10.1007/s10960-004-5797-5 15730067

[6] Palgi S, Klein E, Shamay-Tsoory S. The role of oxytocin in empathy in PTSD. Psychol Trauma. 2017;9(1):70-75. doi:10.1037/tra0000142 27243570

[7] Shalev A, Liberzon I, Marmar C. Post-traumatic stress disorder. N Engl J Med. 2017;376(25):2459-2469. doi:10.1056/NEJMra1612499 28636846

[8] Bisson JI, Cosgrove S, Lewis C, Robert NP. Post-traumatic stress disorder. BMJ. 2015;351:h6161. doi:10.1136/bmj.h6161 26611143 PMC4663500

[9] Chin WS, Shiao JS, Liao SC, Kuo CY, Chen CC, Guo YL. Depressive, anxiety and post-traumatic stress disorders at six years after occupational injuries. Eur Arch Psychiatry Clin Neurosci. 2017;267(6):507-516. doi:10.1007/s00406-016-0762-x 28044191

[10] Usuki M, Matsuoka Y, Nishi D, . Potential impact of propofol immediately after motor vehicle accident on later symptoms of posttraumatic stress disorder at 6-month follow up: a retrospective cohort study. Crit Care. 2012;16(5):R196. doi:10.1186/cc11681 23075426 PMC3682298

[11] Kok BC, Herrell RK, Thomas JL, Hoge CW. Posttraumatic stress disorder associated with combat service in Iraq or Afghanistan: reconciling prevalence differences between studies. J Nerv Ment Dis. 2012;200(5):444-450. doi:10.1097/NMD.0b013e3182532312 22551799

[12] Zatzick DF, Rivara FP, Nathens AB, . A nationwide US study of post-traumatic stress after hospitalization for physical injury. Psychol Med. 2007;37(10):1469-1480. doi:10.1017/S0033291707000943 17559704

[13] Stanley IH. Advancements in the understanding of PTSD and suicide risk: introduction to a special section. Psychol Trauma. 2021;13(7):723-724. doi:10.1037/tra0001121 34723566

[14] Schrader C, Ross A. A review of PTSD and current treatment strategies. Mo Med. 2021;118(6):546-551. 34924624 PMC8672952

[15] Szeszko PR, Lehrner A, Yehuda R. Glucocorticoids and hippocampal structure and function in PTSD. Harv Rev Psychiatry. 2018;26(3):142-157. doi:10.1097/HRP.0000000000000188 29734228

[16] Kida S. Reconsolidation/destabilization, extinction and forgetting of fear memory as therapeutic targets for PTSD. Psychopharmacology (Berl). 2019;236(1):49-57. doi:10.1007/s00213-018-5086-2 30374892 PMC6373183

[17] Sbarski B, Akirav I. Cannabinoids as therapeutics for PTSD. Pharmacol Ther. 2020;211:107551. doi:10.1016/j.pharmthera.2020.107551 32311373

[18] Careaga MBL, Girardi CEN, Suchecki D. Understanding posttraumatic stress disorder through fear conditioning, extinction and reconsolidation. Neurosci Biobehav Rev. 2016;71:48-57. doi:10.1016/j.neubiorev.2016.08.023 27590828

[19] VanElzakker MB, Dahlgren MK, Davis FC, Dubois S, Shin LM. From Pavlov to PTSD: the extinction of conditioned fear in rodents, humans, and anxiety disorders. Neurobiol Learn Mem. 2014;113:3-18. doi:10.1016/j.nlm.2013.11.014 24321650 PMC4156287

[20] Tasbihgou SR, Barends CRM, Absalom AR. The role of dexmedetomidine in neurosurgery. Best Pract Res Clin Anaesthesiol. 2021;35(2):221-229. doi:10.1016/j.bpa.2020.10.002 34030806

[21] Ji MH, Jia M, Zhang MQ, . Dexmedetomidine alleviates anxiety-like behaviors and cognitive impairments in a rat model of post-traumatic stress disorder. Prog Neuropsychopharmacol Biol Psychiatry. 2014;54:284-288. doi:10.1016/j.pnpbp.2014.06.013 25004167

[22] Mo Y, Zimmermann AE. Role of dexmedetomidine for the prevention and treatment of delirium in intensive care unit patients. Ann Pharmacother. 2013;47(6):869-876. doi:10.1345/aph.1AR708 23719785

[23] Lim YP, Yahya N, Izaham A, . The comparison between propofol and dexmedetomidine infusion on perioperative anxiety during regional anesthesia. Turk J Med Sci. 2018;48(6):1219-1227. doi:10.3906/sag-1802-126 30541250

[24] Morena M, Berardi A, Peloso A, . Effects of ketamine, dexmedetomidine and propofol anesthesia on emotional memory consolidation in rats: consequences for the development of post-traumatic stress disorder. Behav Brain Res. 2017;329:215-220. doi:10.1016/j.bbr.2017.04.048 28461010

[25] Dossett LA, Redhage LA, Sawyer RG, May AK. Revisiting the validity of APACHE II in the trauma ICU: improved risk stratification in critically injured adults. Injury. 2009;40(9):993-998. doi:10.1016/j.injury.2009.03.004 19535054 PMC2752660

[26] Vassallo J, Fuller G, Smith JE. Relationship between the Injury Severity Score and the need for life-saving interventions in trauma patients in the UK. Emerg Med J. 2020;37(8):502-507. doi:10.1136/emermed-2019-209092 32748796

[27] Weathers FW, Bovin MJ, Lee DJ, . The Clinician-Administered PTSD Scale for DSM-5 (CAPS-5): development and initial psychometric evaluation in military veterans. Psychol Assess. 2018;30(3):383-395. doi:10.1037/pas0000486 28493729 PMC5805662

[28] American Psychiatric Association. Diagnostic and Statistical Manual of Mental Disorders. 5th ed. American Psychiatric Association; 2013.

[29] Inouye SK, van Dyck CH, Alessi CA, Balkin S, Siegal AP, Horwitz RI. Clarifying confusion: the confusion assessment method. a new method for detection of delirium. Ann Intern Med. 1990;113(12):941-948. doi:10.7326/0003-4819-113-12-941 2240918

[30] Zhong J, Li Y, Fang L, . Effects of sevoflurane and propofol on posttraumatic stress disorder after emergency trauma: a double-blind randomized controlled trial. Front Psychiatry. 2022;13:853795. doi:10.3389/fpsyt.2022.853795 35280171 PMC8914077

[31] Bryant RA. Post-traumatic stress disorder: a state-of-the-art review of evidence and challenges. World Psychiatry. 2019;18(3):259-269. doi:10.1002/wps.20656 31496089 PMC6732680

[32] Joseph NM, Benedick A, Flanagan CD, Breslin MA, Vallier HA. Risk factors for posttraumatic stress disorder in acute trauma patients. J Orthop Trauma. 2021;35(6):e209-e215. doi:10.1097/BOT.0000000000001990 33724967

[33] Gilliam WP, Craner JR, Schumann ME, Gascho K. The mediating effect of pain catastrophizing on PTSD symptoms and pain outcome. Clin J Pain. 2019;35(7):583-588. doi:10.1097/AJP.0000000000000713 30950871

[34] Righy C, Rosa RG, da Silva RTA, . Prevalence of post-traumatic stress disorder symptoms in adult critical care survivors: a systematic review and meta-analysis. Crit Care. 2019;23(1):213. doi:10.1186/s13054-019-2489-3 31186070 PMC6560853

[35] Fu SS, McFall M, Saxon AJ, . Post-traumatic stress disorder and smoking: a systematic review. Nicotine Tob Res. 2007;9(11):1071-1084. doi:10.1080/14622200701488418 17978982

[36] Almeida AN, Tavares C, Tibano A, Sasaki S, Murata KN, Marino R Jr. Dexmedetomidine for awake craniotomy without laryngeal mask. Arq Neuropsiquiatr. 2005;63(3B):748-750. doi:10.1590/S0004-282X2005000500005 16258649

[37] Ibacache ME, Muñoz HR, Brandes V, Morales AL. Single-dose dexmedetomidine reduces agitation after sevoflurane anesthesia in children. Anesth Analg. 2004;98(1):60-63. doi:10.1213/01.ANE.0000094947.20838.8E 14693585

[38] Su X, Meng ZT, Wu XH, . Dexmedetomidine for prevention of delirium in elderly patients after non-cardiac surgery: a randomised, double-blind, placebo-controlled trial. Lancet. 2016;388(10054):1893-1902. doi:10.1016/S0140-6736(16)30580-3 27542303

[39] Hauer D, Ratano P, Morena M, . Propofol enhances memory formation via an interaction with the endocannabinoid system. Anesthesiology. 2011;114(6):1380-1388. doi:10.1097/ALN.0b013e31821c120e 21532463

[40] Lundström S, Twycross R, Mihalyo M, Wilcock A. Propofol. J Pain Symptom Manage. 2010;40(3):466-470. doi:10.1016/j.jpainsymman.2010.07.001 20816571

[41] Soeter M, Kindt M. Noradrenergic enhancement of associative fear memory in humans. Neurobiol Learn Mem. 2011;96(2):263-271. doi:10.1016/j.nlm.2011.05.003 21624479

[42] Ju Q, Xiao Z, Sun W, Zhu M, Lv P. The anesthesia induction effect of dexmedetomidine in patients undergoing laryngeal mask intubation: a systematic review and meta-analysis of 7 RCTs. Ann Palliat Med. 2021;10(12):12358-12366. doi:10.21037/apm-21-2971 35016404

[43] DeViva JC, McCarthy E, Southwick SM, Tsai J, Pietrzak RH. The impact of sleep quality on the incidence of PTSD: results from a 7-year, nationally representative, prospective cohort of U.S. military veterans. J Anxiety Disord. 2021;81:102413. doi:10.1016/j.janxdis.2021.102413 33991819 PMC10693322

[44] McNett S, Lind MJ, Brown RC, . Sleep quality moderates the relationship between anxiety sensitivity and PTSD symptoms in combat-exposed veterans. Behav Sleep Med. 2021;19(2):208-220. doi:10.1080/15402002.2020.1726749 32063030

[45] Williamson JB, Jaffee MS, Jorge RE. Posttraumatic stress disorder and anxiety-related conditions. Continuum (Minneap Minn). 2021;27(6):1738-1763. doi:10.1212/CON.0000000000001054 34881734

[46] Gasperi M, Panizzon M, Goldberg J, Buchwald D, Afari N. Posttraumatic stress disorder and chronic pain conditions in men: a twin study. Psychosom Med. 2021;83(2):109-117. doi:10.1097/PSY.0000000000000899 33337593 PMC7858228

[47] Iljazi A, Ashina H, Al-Khazali HM, . Post-traumatic stress disorder after traumatic brain injury-a systematic review and meta-analysis. Neurol Sci. 2020;41(10):2737-2746. doi:10.1007/s10072-020-04458-7 32415640

