# Supplementary material for: Effect of Dexmedetomidine on Posttraumatic Stress Disorder in Patients Undergoing Emergency Trauma Surgery: A Randomized Clinical Trial
Source: JAMA Netw Open. 2023 Jun 16;6(6):e2318611. doi: 10.1001/jamanetworkopen.2023.18611 (PMC10276303; doi:10.1001/jamanetworkopen.2023.18611)
Supplement: Supplement 1. — Trial Protocol [file jamanetwopen-e2318611-s001.pdf]

Clinical Study Protocol

TITLE: Effect of dexmedetomidine on posttraumatic stress disorder in emergency trauma surgery patients: a randomized controlled trial

PRINCIPAL INVESTIGATOR: Youjia Yu, MD; Yan Li, MD; Rui Yao, MD; Yangzi Zhu, MD.

PROTOCOL SYNOPSIS

**Title:**	Effect of dexmedetomidine on posttraumatic stress disorder in emergency trauma surgery patients: a randomized controlled trial
**Study Type:**	A prospective, Multicenter, randomized, double-blind controlled trial
**Corresponding Author:**	Yangzi Zhu, Jiangsu Province Key Laboratory of Anesthesiology, Xuzhou Medical University; Department of Anesthesiology, Xuzhou Central Hospital, Xuzhou, Jiangsu, China. Rui Yao, Department of Anesthesiology, Xuzhou First People's Hospital, Xuzhou, Jiangsu, China.
**Lead site:**	Xuzhou Central Hospital, Xuzhou Medical University

**Participating centers:**	1. Suzhou Xiangcheng People's Hospital<br>2. Xuzhou Renci Hospital<br>3. Xuzhou First People's Hospital
**Trial Registration:**	Chinese Clinical Trial Register Identifier: ChiCTR2200056162.

I. STUDY OBJECTIVES

PTSD is quite common in people who have suffered trauma, especially those hospitalized for surgery. Dexmedetomidine may reduce or reverse the early consolidation and formation of conditioned fear memory and prevent the occurrence of postoperative PTSD.

The aim of this prospective study was to evaluate the effects of intraoperative and postoperative low-dose intravenous pumping dexmedetomidine or placebo on PTSD among trauma patients undergoing emergency surgery.

II. BACKGROUND

A. Post-traumatic Stress Disorder

Post-traumatic stress disorder (PTSD) is a psychiatric disorder that develops after experiencing major trauma[1], involving a combination of recurring and distressing re-experiencing(e.g., flashbacks, intrusive thoughts), avoidance, negative alterations in mood and cognition, and hyperarousal. Similar or related stimuli may persist for years or decades, with recurrent episodes of traumatic experiences and sustained increases in vigilance and avoidance[2-4]. This mental state is highly debilitating and severely interferes with daily life and social activities[5,6]. Worldwide, the incidence of PTSD can be as high as 10%–22% in recent years, showing a significant upward trend, due to the frequent occurrence of traffic accidents, natural disasters, wars, and terrorist violence[7-9]. Studies have shown that the prevalence of PTSD in the general US population is 6%–8%, and it can be as high as 13%–30% in the military[10,11]. The incidence of PTSD after trauma hospitalization can be as high as 23%[12]. Once PTSD is formed, it can be difficult to manage and is linked to increased risk of suicide, posing a serious burden to the family and society[7,13,14]. Therefore, early and timely intervention for patients with trauma is particularly critical to prevent PTSD.

B. The Pathogenesis of PTSD

The pathogenesis of PTSD is complex, and its exact neurobiological mechanism is still unclear[15]. Studies have shown that recurrent traumatic experience is one of the core symptoms of PTSD, which is found to be closely related to abnormally strengthened fear memory[16,17]. Because of the Pavlovian conditioning principle, environmental information at the time of the trauma, e.g., loud sounds, objects, are associated with the aversive experience (e.g., accident, fall injury). Wounded persons re-exposure to a similar environment may bring back fear memory and lead to physiological and behavioral reactions, which is called fear conditioning[18]. Fear conditioning is pointed out as an outstanding memory feature of PTSD that can explain re-experiencing and, in part, avoidance symptoms[19]. Therefore, intervening in the consolidation and formation of conditioned fear memory is particularly critical to prevent PTSD in patients with trauma in the emergency department, who are in the early stage of fear memory formation and are not yet firmly consolidated.

C. Dexmedetomidine

Dexmedetomidine is widely used in clinical anesthesia to optimize anesthesia and analgesia effects and reduce intraoperative adverse reactions[20]. A preclinical study shows that dexmedetomidine could alleviate anxiety-like behavior and cognitive impairment in PTSD model rats[21]. In clinical studies, the perioperative administration of dexmedetomidine had an anxiolytic effect[22,23]. In another study of conditioned fear memory, dexmedetomidine reduced the strength of the fear memory formed[24]. Therefore, we speculated that dexmedetomidine might attenuate the formation and consolidation of conditioned fear memories early in trauma, thereby preventing the development of PTSD. However, whether dexmedetomidine can reduce the incidence of postoperative PTSD in patients with trauma in the emergency department is still unclear.

III. METHODS

This multicenter trial was conducted at Suzhou Xiangcheng People's Hospital and three other tertiary hospitals in Jiangsu Province, China from January 22 to October 20, 2022. The study protocol was approved by the ethics committees of all participating hospitals and was registered in the Chinese Clinical Trial Registration Center on January 21, 2022. This report follows the Consolidated Standards of Reporting Trials (CONSORT) reporting guideline for randomized studies.

A. Recruiting Methods

Our research team identified potential participants in the emergency room via the Maddie Remote intelligent First Aid platform system. Trauma patients 18 to 60 years old, with American Society of Anesthesiology (ASA) physical status (I&II&III) were eligible for inclusion if they were preparing for emergency surgery. The research centers will obtain permission from each subject to use their protected health information through written authorization.

B. Inclusion Criteria

Trauma patients (car accident, falling, engineering accident, etc.) undergoing emergency surgery aged 18 to 60 years with American Society of Anesthesiologists physical status categories I to III were eligible for inclusion. Non-elderly adults under 60 years were selected for the following reasons: 1. Young people who experience traumatic events are more likely to develop PTSD than elders[25]. 2.

The inclusion of elderly patients will bring some variables related to advanced age, and these variables may have interaction or mediation effects with advanced age, which will reduce the statistical power. 3. For safety reasons, for elderly patients with trauma in the emergency department, it may not be suitable for a long period of pumping dexmedetomidine during the perioperative period. ASA I: physical health, good development and nutrition, normal organ function. ASA II: In addition to the surgical disease, there are mild coexisting diseases and sound functional compensation. ASA III: Severe comorbidity, limited physical activity, but able to cope with daily activities.

C. Exclusion Criteria

1. Craniocerebral or spinal cord injury,hemorrhagic shock decompensation;
2. Liver or renal dysfunction;
3. History of alcohol abuse or drug dependence, history of neurological or psychiatric diseases;
4. Severe visual, hearing or language impairment;
5. History of major physical or mental trauma;
6. Second-degree or third-degree heart blockage, bradyarrhythmia with a baseline rate lower than 50 beats per minute.

D. Consent Procedure

All potential subjects who met the inclusion/exclusion criteria as determined by the chief anesthesiologist or designees were given the opportunity to participate. The family/patients will be obtained to give assent/consent during the screening visit. They will have the opportunity to review consent and ask questions about the study. Families/patients will be asked to summarize in their own words what was involved in the study and how satisfied they were with the risks and benefits of participating. The researcher will also answer any other questions they have before signing the consent form. After signing the consent form, the subject will be provided with a signed and dated copy of the authorization form, and another copy will be placed in the participant's medical record at four hospital centers.

E. Randomization and Blinding

Randomization and blinding

Eligible participants were randomized to either dexmedetomidine or normal saline placebo (control group) using a 1:1 ratio by an online central randomization system. The randomization sequence was based on computer-generated random numbers. Patient clinical management and data collection were sequentially numbered and disclosed by health care practitioners who were not directly involved. Each code was assigned by a random number to one of the two groups: the placebo group or the dexmedetomidine group. An anesthetic nurse who was not otherwise involved in the study prepared dexmedetomidine and normal saline in advance, and they were each kept in syringes labeled only with the patient number. The surgeons, study statistician personnel, research staff who assessed the outcomes, and patients themselves were blinded to the treatment group.

F. Sample Size Calculation

According to the literature, the incidence of PTSD among trauma patients 1 month postoperatively was 23.2%. Our pilot study showed that 11.1% of patients with trauma in the emergency department who received dexmedetomidine during surgery developed PTSD 1 month postoperatively. Hence, this trial required 152 patients in each group with a power of 80% at a significance of $\alpha = 0.05$. We decided to recruit 350 patients (with 175 in each group) considering a possible dropout rate of 15%.

G. Statistical Analysis

The outcome analyses were performed in the modified intention-to-treat population. The data were analyzed using SPSS statistical software version 23.0 (IBM). The Kolmogorov–Smirnov test was used to determine whether the continuous data conformed to the normal distribution. Continuous variables were presented as the mean ± standard deviation or medians (Inter quartile range, IQR). The continuous data with normal distribution were compared with the independent-sample t test. The continuous data with nonnormal distribution were analyzed using the Kruskal–Wallis rank-sum test. Associations between categorical variables were assessed using the $\chi 2$ test or Fisher exact test. The odds ratio (OR) and 95% confidence interval (CI) were calculated to analyze the effect of dexmedetomidine on the prevention of PTSD in the primary outcome. The association between the primary outcome and intervention were adjusted for some potential confounding using binary logistic regression, including age, sex, smoking, trauma time, ISS, APACHE II, ICU admission, type of surgery, study sites and duration of surgery. The odds ratio (OR) and 95% confidence interval (CI)

were calculated to analyze the association between dexmedetomidine dose and occurrence of PTSD in the post hoc analyses. The association between dexmedetomidine dose and occurrence of PTSD were adjusted for some potential confounding using binary logistic regression, including age, sex, smoking, trauma time, ISS, APACHE II, ICU admission, type of surgery, study sites and duration of surgery. Repeated measures of continuous variables at different time in secondary outcomes were analyzed using repeated measures analysis of variance (ANOVA). Spearman's correlation test was used to analyze the correlation between dexmedetomidine dose and CAPS-5 score in the post hoc analysis. P value <0.05 indicated a statistically significant association.

H. Interventions

Dexmedetomidine or placebo (normal saline) was administered at a maintenance dose of 0.1 μg/(kg · h) from the start of anesthesia until the end of surgery and at the same rate after surgery from 9 p.m. to 7 a.m. on days 1–3.

I. Administration Method

Based on previous studies and a pilot study, dexmedethidine infused at a dose of 0.1μg/kg per hour (maintained during surgery and from 9 pm to 7 am on postoperative days 1-3) can reduce perioperative anxiety symptoms and significantly improve sleep quality without significant circulatory effects. The pump protocol has good relative compliance. For the above reasons, we finally selected the low-dose dexmedetomidine pump injection scheme.

J. Management of the perioperative period

1. Preoperative assessment

During the preoperative assessment, we collected baseline data include demographic characteristics, past history and American Society of Anesthesiologists (ASA) classification. Acute physiology and chronic health Evaluation Ⅱ (APACHE Ⅱ) and injury severity score (ISS) were used to assess the severity of trauma. And we obtained the informed consent of patients or family members if eligible.

2. Management of premedication

The study medication (dexmedetomidine hydrochloride, 200 μg/2 mL, and normal saline, 2 mL) was provided and assigned on the basis of randomization by an anesthetic nurse who was not involved in the rest of the study. The drug was diluted to 50 mL with normal saline before administration (i.e., dexmedetomidine hydrochloride at a final concentration of 4μg/mL). Dexmedetomidine or placebo (normal saline) was administered at a maintenance dose of 0.1μg/kg per hour from the start of anesthesia until the end of surgery and at the same rate after surgery from 9 p.m. to 7 a.m. on days 1 through 3. The infusion of dexmedetomidine or placebo was suspended or permanently stopped during or after the operation according to the patients' own condition or other objective factors. The dose of infusion was recorded.

3. Management of general anesthesia

Intraoperative monitoring included non-invasive blood pressure, electrocardiogram (ECG), SpO2, radial artery blood pressure and nasopharyngeal temperature. Intravenous anesthesia induction was performed using midazolam, sufentanil, etomidate and rocuronium. After successful tracheal intubation, the patient was mechanically ventilated and end-tidal carbon dioxide (PETCO2) was maintained between 35 and 45 mmHg. Anesthesia was maintained using propofol, remifentanil and cisatracurium to keep a bispectral index values of 40 to 60. Hypotension (mean arterial pressure <65 mmHg or a decrease of 20% from baseline) and bradycardia (heart rate <50 beats/min) were treated. A thermal blanket was used during the operation to maintain nasopharyngeal temperature above 36 ℃.

4. Postoperative Analgesia

PCIA was used in both groups. Postoperative analgesia was achieved with 3 μg/kg of sufentanil and 20 mg of azasetron in 100 mL of normal saline. The background infusion rate was 2 mL/h. After returning to the ward, if the VAS pain score＞4, intravenous flurbiprofen axetil 100mg.

5. Management of hypotension and bradycardia

When the mean arterial pressure is lower than 65, fluid infusion or vasoactive drugs should be used. Atropine 0.5mg if heart rate is less than 45 beats per minute; If more than two treatments were performed during the operation or on the same night, the pump speed of dexmedetamidine (normal saline) was reduced (intraoperative) or the pump was stopped. Depending on the patient's daytime condition, if daytime hypotension or a heart rate of less than 45 beats per minute occurred for two

consecutive days, dexmedetomidine pumping was permanently discontinued during the following night.

K. Surgical anatomical sites

Intestines, liver, spleen, limb, lungs, ribs or diaphragm.

L. Outcome measures:

The primary outcome was the occurrence of PTSD. It was assessed with the Clinician-Administered PTSD Scale for DSM-5 (CAPS-5) 1 month after surgery. The CAPS-5 score (derived from the CAPS-5 scale) was used to evaluate PTSD severity. At the beginning of the study, our research team used CAPS-5 score and PTSD Checklist for DSM-5 (PLC-5) score simultaneously to evaluate trauma patients. As participants generally reported that the evaluation time was too long and their cooperation was not good, we finally selected CAPS-5 score for evaluation after the approval of the Ethics Committee. Professionally trained doctors, blinded to treatment group assignments, carried out the diagnostic assessments at both times in tranquil surroundings. The CAPS-5 is a structured diagnostic interview and considered the gold standard in PTSD evaluation. The CAPS-5 provides a continuous measure of the severity of overall PTSD and of the four symptom clusters (intrusions, avoidance, negative alterations in cognition/mood, arousal and reactivity) and presence/absence of PTSD diagnosis, which can be administered by appropriately trained paraprofessionals. The diagnosis requirement can be summarized as an exposure to a stressor that is accompanied by at least one intrusion symptom, one avoidance symptom, two negative alterations in cognitions and mood symptoms, and two arousal and reactivity turbulence symptoms, persisting for at least one month, with functional impairmen.

The secondary outcomes included postoperative (24h, 48h and 1month) pain using the Visual Analogue Scale (VAS), Postoperative delirium using the confusion assessment method criteria (measured twice daily for 3 days) , nausea, itching, subjective sleep quality (measured for 3 days) using Numerical Rating Scale (NRS), anxiety (measured for 3 days) using Beck Anxiety Inventory (BAI), and occurrence of adverse events (including hypertension, hypotension, bradycardia, tachycardia, hypoxemia, and other complications such as cerebrovascular events, myocardial infarction, heart failure, and acute kidney injury).

IV. SAFETY CONCERNS

Dexmedetomidine is becoming increasingly popular as it promotes a natural, non rapid-eye-movement sleep, anxiolysis, and analgesia, without concurrent respiratory depression. Hypotension and bradycardia are potential hemodynamic side effects of dexmedetomidine, but most studies found these effects to be self-limiting and clinically benign. Generally, dexmedetomidine has been administered to millions of patients in various surgical procedures every year for at least 20 years, with a good safety profile in the perioperative use and use for the intensive care. To maximize safety, strict screening criteria were used to exclude patients with hemodynamic instability, bradycardia, or second degree or greater block, and low-dose dexmedetomidine (0.1μg/kg per hour) was administered. To maximize safety, strict screening criteria were used to exclude patients with hemodynamic instability, bradycardia, or second degree or greater block, and a low-dose of dexmedetomidine (0.1ug/kg/hour) was administered. We also implemented a dual management plan for potential risks (**J. Management of the perioperative period**) to ensure patient safety.

V. NONCOMPLIANCE RATE REPORT AND IMPROVEMENT

The rate of non-compliance was 20.1% in the dexmedetomidine group and 24.4% in the control group(please see them in eTable2). The primary reasons were as follows: 1. To maximize safety, infusion of dexmedetomidine (or normal saline) was discontinued in patients who had two decreases in blood pressure or a heart rate of less than 45 beats per minute that required medical management one night (the first day included the daytime intraoperative period). Depending on the patient's daytime condition, if daytime hypotension or a heart rate of less than 45 beats per minute occurred for two consecutive days, dexmedetomidine pumping was permanently discontinued the following night. 2. Some patients did not return to the ward before 9 PM for intervention; 3. Some patients' family members turned off the infusion pump, due to equipment error alarm. At first, for safety reasons, the researchers informed the patients and their families that they could immediately turn off the infusion pump and call the nurse if they had discomfort symptoms such as palpitations and chest tightness. In the later stage, we made improvements, and informed the patients and their families that if they had discomfort symptoms such as palpitation and chest tightness, they should call the nurses, and refrain from turning off the infusion pump by themselves.

VI. DATE AND SAFETY MONITORING

Handle and follow up until properly solved or the condition is stable, and timely report serious adverse events and accidents to the ethics committee, competent authorities and drug regulatory authorities as required; The principal investigators performed a cumulative review of all adverse events at regular intervals and convened investigators' meetings as necessary to assess the risks and benefits of the study. This study was a double-blind trial, and blinding was opened if necessary to ensure the safety and rights of the subjects. An independent data monitor will be assigned to monitor the study data, and an independent data and safety monitoring board will be established for high-risk studies to monitor accumulated safety and efficacy data to determine whether to continue the study. Clinical research will develop corresponding data safety monitoring programs according to the risk level.

VII. FUNDING

This clinical research received National Natural Science Foundation of China (81801332); Science and Technology Developnent Plan Project of Suzhou(SKJYD2021035); Science and Technology Development Plan Project of Suzhou(SKJYD2022078).

VIII. INFORMATION CONFIDENTIALITY

The medical records were kept at the hospital and were accessible to the investigators and the ethics committee, which had access to the patient's medical records. Any public reporting of the results of this study will not reveal the individual identity of the patients. The patient's personal information and medical records are kept strictly confidential.

IX. REFERENCES

Hori H, Kim Y. Inflammation and post-traumatic stress disorder. Psychiatry Clin Neurosci. 2019;73(4):143-153. doi:10.1111/pcn.12820.

2. Koirala R, Søegaard EGI, Thapa SB. Updates on Pharmacological Treatment of Post-Traumatic Stress Disorder. JNMA J Nepal Med Assoc. 2017;56(206):274-280. doi:10.31729/jnma.3108.

3. He M, Wei JX, Mao M, et al. Synaptic Plasticity in PTSD and associated Comorbidities: The Function and Mechanism for Diagnostics and Therapy. Curr Pharm Des. 2018;24(34):4051-4059. doi:10.2174/1381612824666181120094749.

4. Qi W, Gevonden M, Shalev A. Prevention of Post-Traumatic Stress Disorder After Trauma: Current Evidence and Future Directions. Curr Psychiatry Rep. 2016;18(2):20. doi:10.1007/s11920-015-0655-0.

5. McCleery JM, Harvey AG. Integration of psychological and biological approaches to trauma memory: implications for pharmacological prevention of PTSD. J Trauma Stress. 2004;17(6):485-496. doi:10.1007/s10960-004-5797-5.

6. Palgi S, Klein E, Shamay-Tsoory S. The role of oxytocin in empathy in PTSD. Psychol Trauma. 2017;9(1):70-75. doi:10.1037/tra0000142.

7. Shalev A, Liberzon I, Marmar C. Post-Traumatic Stress Disorder. N Engl J Med. 2017; 376 (25):2459-2469. doi:10.1056/NEJMra1612499.

8. Bisson JI, Cosgrove S, Lewis C, Robert NP. Post-traumatic stress disorder. BMJ. 2015;351:h6161. doi: 10.1136/bmj.h6161.

9. Chin WS, Shiao JS, Liao SC, Kuo CY, Chen CC, Guo YL. Depressive, anxiety and post-traumatic stress disorders at six years after occupational injuries. Eur Arch Psychiatry Clin Neurosci. 2017;267(6):507-516. doi:10.1007/s00406-016-0762-x.

10. Usuki M, Matsuoka Y, Nishi D, et al. Potential impact of propofol immediately after motor vehicle accident on later symptoms of posttraumatic stress disorder at 6-month follow up: a retrospective cohort study. Crit Care. 2012;16(5):R196. doi:10.1186/cc11681.

11. Kok BC, Herrell RK, Thomas JL, Hoge CW. Posttraumatic stress disorder associated with combat service in Iraq or Afghanistan: reconciling prevalence differences between studies. J Nerv Ment Dis. 2012;200(5):444-450. doi:10.1097/NMD.0b013e3182532312.

12. Zatzick DF, Rivara FP, Nathens AB, et al. A nationwide US study of post-traumatic stress after hospitalization for physical injury. Psychol Med. 2007;37(10):1469-1480. doi:10.1017/S0033291707000943.

13. Stanley IH. Advancements in the understanding of PTSD and suicide risk: Introduction to a special section. Psychol Trauma. 2021;13(7):723-724. doi:10.1037/tra0001121.

14. Schrader C, Ross A. A Review of PTSD and Current Treatment Strategies. Mo Med. 2021;118(6):546-551.

15. Szeszko PR, Lehrner A, Yehuda R. Glucocorticoids and Hippocampal Structure and Function in PTSD. Harv Rev Psychiatry. 2018;26(3):142-157. doi:10.1097/HRP.0000000000000188.

16. Kida S. Reconsolidation/destabilization, extinction and forgetting of fear memory as therapeutic targets for PTSD. Psychopharmacology (Berl). 2019;236(1):49-57. doi:10.1007/s00213-018-5086-2.

17. Sbarski B, Akirav I. Cannabinoids as therapeutics for PTSD. Pharmacol Ther. 2020;211:107551. doi:10.1016/j.pharmthera.2020.107551.

18. Bowers ME, Ressler KJ. An Overview of Translationally Informed Treatments for Posttraumatic Stress Disorder: Animal Models of Pavlovian Fear Conditioning to Human Clinical Trials. Biol Psychiatry. 2015;78(5):E15-E27. doi:10.1016/j.biopsych.2015.06.008.

19. Paredes Molina CS, Berry S, Nielsen A, Winfield R. PTSD in civilian populations after hospitalization following traumatic injury: A comprehensive review. Am J Surg. 2018;216(4):745-753. doi:10.1016/j.amjsurg.2018.07.035.

20. Tasbihgou SR, Barends CRM, Absalom AR. The role of dexmedetomidine in neurosurgery. Best Pract Res Clin Anaesthesiol. 2021;35(2):221-229. doi:10.1016/j.bpa.2020.10.002.

21. Ji MH, Jia M, Zhang MQ, et al. Dexmedetomidine alleviates anxiety-like behaviors and cognitive impairments in a rat model of post-traumatic stress disorder. Prog Neuropsychopharmacol Biol Psychiatry. 2014;54:284-288. doi:10.1016/j.pnpbp.2014.06.013.

22. Mo Y, Zimmermann AE. Role of dexmedetomidine for the prevention and treatment of delirium in intensive care unit patients. Ann Pharmacother. 2013;47(6):869-876. doi:10.1345/aph.1AR708.

23. Lim YP, Yahya N, Izaham A, et al. The comparison between propofol and dexmedetomidine infusion on perioperative anxiety during regional anesthesia. Turk J Med Sci. 2018;48(6):1219-1227. doi:10.3906/sag-1802-126.

24. Morena M, Berardi A, Peloso A, et al. Effects of ketamine, dexmedetomidine and propofol anesthesia on emotional memory consolidation in rats: Consequences for the development of post-traumatic stress disorder. Behav Brain Res. 2017;329:215-220. doi:10.1016/j.bbr.2017.04.048.

25. Karam EG, Friedman MJ, Hill ED, et al. Cumulative traumas and risk thresholds: 12-month PTSD in the World Mental Health (WMH) surveys. Depress Anxiety. 2014;31(2):130-142. doi:10.1002/da.22169.
