# Supplementary material for: Effect of Dexmedetomidine on Posttraumatic Stress Disorder in Patients Undergoing Emergency Trauma Surgery: A Randomized Clinical Trial
Source: JAMA Netw Open. 2023 Jun 16;6(6):e2318611. doi: 10.1001/jamanetworkopen.2023.18611 (PMC10276303; doi:10.1001/jamanetworkopen.2023.18611)
Supplement: Supplement 2. — eTable 1. Patient Characteristics and Perioperative Data eTable 2. Safety Outcomes Among the Two Groups eTable 3. Association Between Dexmedetomidine Dose and Occurrence of PTSD in the Dexmedetomidine Group eFigure 1. Post Hoc Analysis of Participants Who Were Compliant With Pumping Protocol to Compare the Incidence of PTSD Among 2 Groups eFigure 2. Relationship Between Dexmedetomidine Dose and CAPS-5 Score [file jamanetwopen-e2318611-s002.pdf]

Supplementary Online Content

Yu Y, Li Y, Han D, et al. Effect of dexmedetomidine on posttraumatic stress disorder in patients undergoing emergency trauma surgery: a randomized clinical trial. *JAMA Netw Open*. 2023;6(6):e2318611. doi:10.1001/jamanetworkopen.2023.18611

eTable 1. Patient Characteristics and Perioperative Data
eTable 2. Safety Outcomes Among the Two Groups
eTable 3. Association Between Dexmedetomidine Dose and Occurrence of PTSD in the Dexmedetomidine Group
eFigure 1. Post Hoc Analysis of Participants Who Were Compliant With Pumping Protocol to Compare the Incidence of PTSD Among 2 Groups
eFigure 2. Relationship Between Dexmedetomidine Dose and CAPS-5 Score

This supplementary material has been provided by the authors to give readers additional information about their work.

© 2023 Yu Y et al. *JAMA Network Open.*

eTable 1. Patient Characteristics and Perioperative Data

Characteristics	Patients, No. (%) (N = 350) Normal saline (n = 175)	Dexmedetomidine (n = 175)	*P* value
Sex			
Female	72 (41.1)	64 (36.6)	0.38
Male	103 (59.7)	111 (63.4)	
Age, mean (SD), y	39.4(10.3)	41.0(10.0)	0.14
Body mass indexa, mean (SD)	25.3(3.6)	26.0(4.9)	0.11
American Society of Anesthesiologists physical status category			
I	120 (68.6)	106(60.6)	0.23
II	34 (19.4)	47(26.9)	
III	21(12.0)	22(12.5)	
Hypertension	31 (17.7)	24 (13.7)	0.30
Diabetes	22 (12.6)	28(16.0)	0.36
Smoking	35 (20.0)	29 (16.6)	0.41
APACHE II, median (IQR)	7.4 (5.8-9.4)	7.0 (5.0-9.0)	0.22
ISS, median (IQR)	14.6 (7.1-19.8)	14.1 (9.2-19.7)	0.77
Anatomical site of surgery			
Intestines	44 (25.1)	34 (19.4)	
Liver/spleen	20(11.4)	31 (17.7)	0.28
Limb	83 (47.4)	85 (48.6)	
Lungs/ribs/diaphragm	28 (16.0)	23 (13.1)	
Trauma timeb, median (IQR), min	100 (85-120)	95 (90-120)	0.70
Surgery time, median (IQR), min	95 (80-120)	95 (75-120)	0.59
Delayed emergence from anesthesia	8(4.6)	12(6.9)	0.34
Intraoperative know	0	0	-
Blood loss, median (IQR), mL	525 (275-700)	500(300-700)	0.74
Blood transfusion	30(17.1)	32 (18.3)	0.78
ICU admission	16 (9.1)	14 (8.0)	0.70

Abbreviations: APACHE II, Acute Physiology and Chronic Health Evaluation II; ISS, injury severity score.

[a] Body mass index is calculated as weight in kilograms divided by height in meters squared.

[b] Trauma time was the time from calling the emergency calls to anesthesia induction.

© 2023 Yu Y et al. *JAMA Network Open.*

eTable 2. Safety Outcomes Among the Two Groups

	Normal saline (n = 154)	Dexmedetomidine (n = 156)	χ2	*P* value
Adverse events				
Bradycardia	20	28	1.46	0.23
Hypotension	32	39	0.78	0.38
Tachycardia	21	11	3.63	0.06
Hypertension	10	5	1.82	0.18
Hypoxaemia	2	0	2.04	0.25
Modification of infusion				
None[a]	123	118	0.80	0.37
Infusion stopped temporarily[b]	5	8	NA	NA
Infusion stopped permanently[b]	3	2	NA	NA
Infusion rate decreased[b]	2	3	NA	NA
Others[b]	21	25	NA	NA

Abbreviations: NA, not applicable.

[a] Complied with the pumping protocol.

[b] Did not comply with the pumping protocol.

© 2023 Yu Y et al. *JAMA Network Open.*

eTable 3. Association Between Dexmedetomidine Dose and Occurrence of PTSD in the Dexmedetomidine Group

**Facts**	***B***	**SE**	***Wald***	**OR**	**95%*CI***	***P* value**
Dex dose	-0.10	0.05	5.28	0.90	0.82-0.99	0.02
Age	0.00	0.03	0.03	1.00	0.95-1.06	0.88
Sex[a]	-0.65	0.61	1.11	0.52	0.16-1.74	0.29
ISS	-0.06	0.05	1.39	0.95	0.86-1.04	0.24
APACHE II	0.02	0.10	0.06	1.03	1.03-0.85	0.80
Trauma time	-0.01	0.01	0.99	0.99	0.96-1.01	0.32
Surgery time	-0.00	0.01	0.05	1.00	0.98-1.01	0.82
Smoking[b]	0.03	0.69	0.00	1.03	0.27-4.01	0.96
Surgery type	NA	NA	4.83	NA	NA	0.19
Surgery type(1)[c]	-1.06	0.82	1.65	0.35	0.13-2.73	0.20
Surgery type(2)[c]	-1.46	0.68	4.65	0.23	0.08-1.02	0.03
Surgery type(3)[c]	-1.20	0.87	1.88	0.30	0.06-1.67	0.17
ICU admission[d]	2.96	1.08	7.55	19.30	2.34-159.47	0.01
Study sites	NA	NA	5.19	NA	NA	0.16
Study sites(1)[e]	0.84	0.60	1.94	2.32	0.08-1.02	0.16
Study sites(2)[e]	0.43	0.87	0.24	1.53	0.08-1.02	0.62
Study sites(3)[e]	-1.83	1.29	2.00	0.16	0.08-1.02	0.16

Abbreviations: Dex, Dexmedetomidine ; APACHE II, Acute Physiology and Chronic Health Evaluation II; ISS, injury severity score; NA, not applicable; ICU, intensive care unit.

[a] Males were used as the reference.

[b] Non-smoking was used as the reference.

[c] The surgical site was the intestine as the reference.

[d] No ICU admission was used as the reference.

[e] Xuzhou Renci hospital was used as the reference.

© 2023 Yu Y et al. *JAMA Network Open.*

eFigure 1. Post Hoc Analysis of Participants Who Were Compliant With Pumping Protocol to Compare the Incidence of PTSD Among 2 Groups

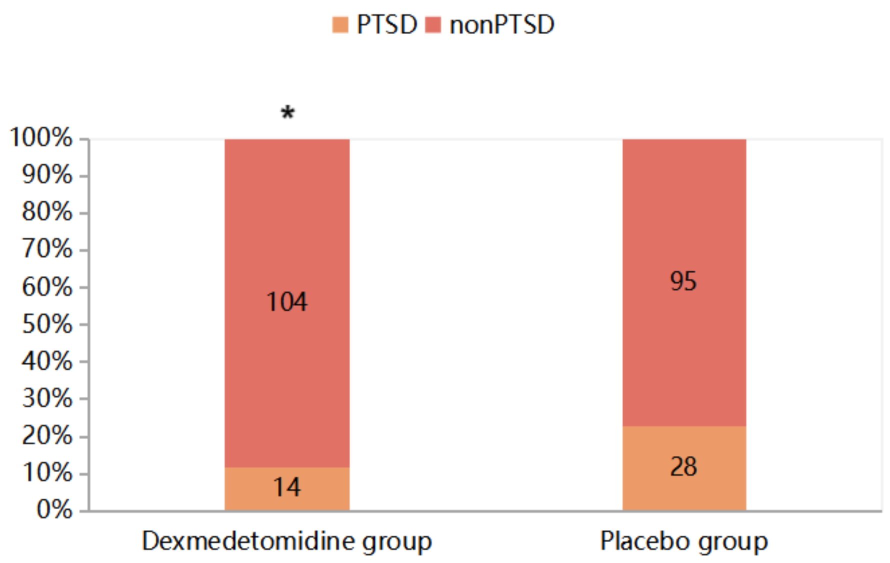

Abbreviations: PTSD, post-traumatic stress disorder

* $P < 0.05$ compared with the placebo group.

© 2023 Yu Y et al. *JAMA Network Open.*

eFigure 2. Relationship Between Dexmedetomidine Dose and CAPS-5 Score

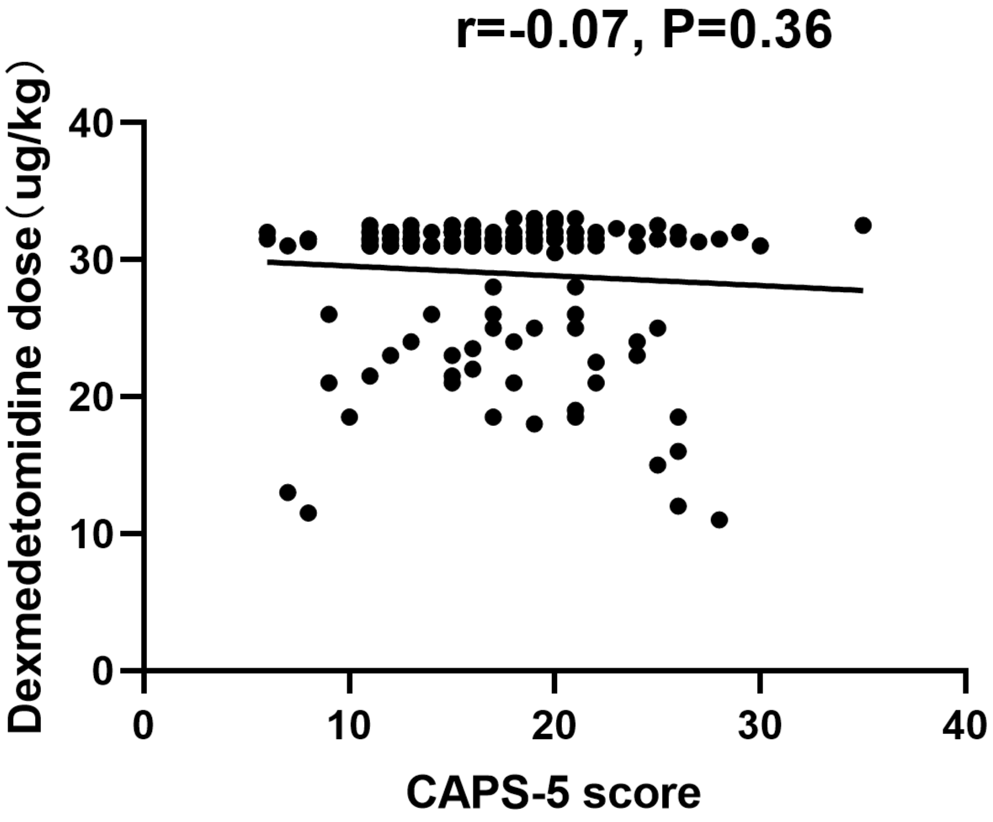

Abbreviations: CAPS-5, Clinician-Administered PTSD Scale for DSM-5.

© 2023 Yu Y et al. *JAMA Network Open.*
