# Supplementary material for: Effect of Dexmedetomidine on Posttraumatic Stress Disorder in Patients Undergoing Emergency Trauma Surgery: A Randomized Clinical Trial
Source: JAMA Netw Open. 2023 Jun 16;6(6):e2318611. doi: 10.1001/jamanetworkopen.2023.18611 (PMC10276303; doi:10.1001/jamanetworkopen.2023.18611)
Supplement: Supplement 3. — Data Sharing Statement [file jamanetwopen-e2318611-s003.pdf]

Data Sharing Statement

Yu. Effect of Dexmedetomidine on Posttraumatic Stress Disorder in Patients Undergoing Emergency Trauma Surgery. *JAMA Netw Open*. Published June 16, 2023.
doi:10.1001/jamanetworkopen.2023.18611

Data
Data available: Yes
Data types: Deidentified participant data
How to access data: zhuyz@188.com or yaorui_edu@163.com
When available: With publication

Supporting Documents
Document types: Statistical/analytic code
How to access documents: zhuyz@188.com or yaorui_edu@163.com
When available: With publication

Additional Information
Who can access the data: researchers whose proposed use of the data has been approved
Types of analyses: for a specified purpose
Mechanisms of data availability: with investigator support
